# Characteristics of Obstructive Sleep Apnea Patients With Hypertension and Factors Associated With Autotitration Acceptance

**DOI:** 10.3389/fpsyt.2021.706275

**Published:** 2022-01-04

**Authors:** Xuan Zhang, Ning Zhang, Yang Yang, Shuo Wang, Ping Yu, Bo-Yuan Guan, Chun-Xue Wang

**Affiliations:** ^1^Department of Neurology, Beijing Tian Tan Hospital, Capital Medical University, Beijing, China; ^2^Department of Neuropsychiatry and Behavioral Neurology and Clinical Psychology, Beijing Tian Tan Hospital, Capital Medical University, Beijing, China; ^3^China National Clinical Research Center for Neurological Diseases, Beijing, China; ^4^Department of Clinical Psychology, Capital Medical University, Beijing, China; ^5^Beijing Institute of Brain Disorders, Beijing, China

**Keywords:** obstructive sleep apnea, hypertension, titration test, sleep efficiency, apnea hypopnea index

## Abstract

In order to explore the characteristics and treatment status of obstructive sleep apnea (OSA) patients with hypertension, a retrospective study was conducted on 306 patients admitted from October 2018 to December 2019. According to the apnea hypopnea index (AHI), OSA patients with hypertension were divided into three groups. 69 cases were mild OSA (5 ≤ AHI < 15), 86 cases were moderate (15 ≤ AHI < 30), and 151 cases were severe (AHI ≥ 30). Compared with patients in the mild and moderate groups, the severe group had more male patients, with higher body mass index (BMI) and non-rapid eye movement stage 1 accounted for total sleep time (N1%), and lower non-rapid eye movement stage 2 accounted for total sleep time (N2%), average and minimum blood oxygen. Among all the patients, those who underwent the titration test accounted for 20.6% (63/306). Multivariate analysis showed that sleep efficiency (*p* < 0.001) and AHI (*p* < 0.001) were independent factors for patients to accept titration test. OSA patients with hypertension had a low acceptance of titration therapy. These people with higher sleep efficiency and AHI were more likely to receive autotitration.

## Introduction

Sleep apnea hypopnea syndrome has a strong correlation with hypertension. Studies have found that more than 20% of hypertension patients have obstructive sleep apnea (OSA), and the prevalence of hypertension in OSA patients exceeds 50% (1–3). Hypertension patients with OSA are mostly refractory, and the effect of antihypertensive drugs alone is not good, and there is no effective evidence to prove that antihypertensive drugs can affect the severity of OSA patients. Therefore, such patients need to apply continuous positive airway pressure (CPAP) at the same time (4–6). Meta-analysis and randomized controlled trials have shown that CPAP can lower the blood pressure of OSA patients by about 2 mmHg. Although the effect of lowering blood pressure is relatively mild, it can still have a beneficial effect on the prognosis of cardiovascular disease outcomes (7–11). For adult patients with OSA and hypertension, the benefits of using CPAP therapy may outweigh the potential harm and burden caused by no intervention (12).

CPAP is usually set at a fixed pressure to prevent the upper airway from collapsing. The reference standard for determining the fixed pressure is the nocturnal CPAP titration study with polysomnography (PSG) monitoring in the sleep laboratory. In the past, manual titration was often used, but this method was time-consuming and expensive (13). A new type of automatic positive airway pressure (APAP) device can automatically adjust the pressure according to the physiological signals measured by the instrument. This method can reduce costs and improve the availability of treatment (14). Compared with the traditional CPAP titration method, OSA patients who took the APAP titration clinical pathway have the same or even better compliance with CPAP treatment (15, 16).

However, the reception and use of CPAP therapy is not ideal. Only 27.9% of OSA patients who had been prescribed CPAP received the treatment (17). The acceptance rate for elderly patients is 31.5% (18) and that of moderate to severe patients is 57.8% (19). Therefore, the acceptance and compliance of CPAP treatment has become a key issue for this group of people. At present, research on subjects such as OSA patients combined with hypertension in China is still very limited.

This study retrospectively analyzed 306 OSA patients with hypertension from October 2018 to December 2019. The purpose of the study is to investigate the clinical characteristics of patients with OSA and hypertension and the acceptance status of the titration test.

## Materials and Methods

### Study Population

This was a retrospective case series of patients diagnosed with OSA and hypertension at the Sleep Center of Beijing Tiantan Hospital between October 2018 to December 2019. A total of 306 patients were included. OSA is defined as patients who reported apnea hypopnea index (AHI) ≥5/h after completing PSG (20), and hypertension is defined as having been diagnosed by a doctor and recommended for medical treatment. The patients included in the study had no other comorbidities. All the patients completed polysomnography (PSG) on the first night, and then people who accepted the titration test would be scheduled for the APAP titration test with an oral-nasal mask on the second night. Both tests were done in the sleep laboratory for full-night. APAP pressure setting was based on the results of the previous night's PSG report to get an appropriate range. The research protocol was approved by the Medical Ethics Committee of Beijing Tiantan Hospital and the Medical Ethics Committee of Capital Medical University.

### Scale Evaluation

Before starting PSG, each participant completed baseline information, medical history, and related scale evaluations, including Insomnia Severity Index (ISS) and Epworth Sleepiness Scale (ESS), Montreal Cognitive Assessment (MoCA), Hamilton Anxiety Rating Scale (HAMA), and Hamilton Depression Rating Scale-17 (HAMD). The scale evaluation is a routine examination of the patients in the sleep center, which was completed by professional clinicians, and all the patients' informed consent had been obtained. Other information could be obtained from the patients' medical records.

### PSG

The sleep technicians conducted at least 6 ho of standard nocturnal polysomnography research. The standard PSG record includes 6 electroencephalogram (EEG) leads (F3, F4, C3, C4, O1, and O2), 1 electrocardiograph (ECG) channel, 2 electrooculogram channels, 4 electromyography channels (bilateral jaws and bilateral lower limbs), video surveillance, thoracic breathing channel, abdominal breathing channel, the airflow of the nose and mouth is recorded by the thermal sensor, the position change is recorded by the position sensor, and the oxygen saturation is recorded by the fingertip oximeter. All the PSG data were confirmed by polysomnography technology experts according to the American Academy of Sleep Medicine 2007 standard. Obstructive apnea is defined as the need to reduce the airflow by 80–100% for at least 10 s, and the continuous breathing effort is recorded in the chest and abdominal movement channels. AHI is calculated as the total number of apneas and hypopneas during sleep per hour. The definition of sleep efficiency is the ratio of total sleep time to total recording time. For OSA patients with hypertension, sleep specialists will recommend them complete the APAP titration test in the laboratory, and let them understand the basic principles, benefits and possible side effects of CPAP.

### Statistical Analysis

Use SPSS22.0 for data collection and statistical analysis. The comparison between the two groups uses independent sample *t*-test (when the continuous variable obeys the normal distribution) and the Mann-Whitney U test (when the continuous variable obeys the non-normal distribution); the comparison among three groups uses analysis of variance (when the continuous variable obeys a normal distribution) and Kruskal-Wallis H test (when the continuous variable obeys a non-normal distribution), the Bonferroni method is used for the post-test. The chi-square test was used to compare categorical variables between groups. Spearman rank test was used for correlation analysis. All variables related to the acceptance of titration test are introduced into the multiple logistic regression model (forward, conditional). The multivariate odds ratio (OR) and 95% confidence interval (CI) are given. A bilateral *P*-value < 0.05 was considered statistically significant.

## Results

### Overall Demographic Data and Polysomnography Parameters

Table 1 shows the overall characteristics of the 306 OSA patients with hypertension included in this study. There were 213 males and 93 females. The average age of the patients was 55.8 ± 12.9 years. All patients had symptoms such as snoring, daytime sleepiness, cognitive decline or depression in varying degrees. 42% of patients have periodic limb movements.

**Table 1:** Characteristics of the patients according to the severity of OSA.

**Characteristics**	**Total cohort**	**5 ≤ AHI < 15**	**15 ≤ AHI < 30**	**AHI ≥ 30**	***p*-value**
	**(*n =* 306)**	**(*n =* 69)**	**(*n =* 86)**	**(151)**	
**Demographic and clinical characteristics**					
% of total	100	22.5	28.1	49.3	
Age, y	55.8 ± l2.9	56.5 ± l2.6	57.5 ± l1.9	54.5 ± l3.4	0.185
Male, *n* (%)	213 (69.6)	38 (55.1)	49 (57)	126 (69.6)	**<0.001**
BMI, kg/m^2^	27.1 ± 4.3	25.8 ± 3.6	26.2 ± 3.7	28.1 ± 4.6	**<0.001**
Tobacco use, *n* (%)	139 (45.4)	29 (42)	32 (37.2)	78 (51.7)	0.081
Alcohol drinking, *n* (%)	170 (55.6)	34 (49.3)	41 (47.7)	95 (62.9)	**0.037**
**Polysomnographic data**					
Sleep efficiency,%	70.3 ± 16.5	72.7 ± 16.4	68.2 ± 17.7	70.5 ± 15.8	0.185
**Sleep stage,%TST**					
REM	15.7 ± 6.8	17.5 ± 7.4	15.3 ± 6.9	15.2 ± 6.3	0.084
N1	25 ± 17.1	18 ± 11.2	21.3 ± 15.1	30.3 ± 18.8	**<0.001**
N2	54.2 ± 15.3	59 ± 12.9	56.8 ± 12.2	50.5 ± 17	**<0.001**
N3	5 ± 6.2	5.5 ± 5.8	6.6 ± 7.9	4 ± 4.9	**0.019**
Mean SpO_2_,%	94.6 ± 2.9	95.6 ± 1.4	95.3 ± 3	93.8 ± 3.1	**<0.001**
Lowest SpO_2_,%	81.4 ± 9.6	87 ± 5.1	85.4 ± 5.9	76.7 ± 10.4	**<0.001**
**Scale**					
ISI	9.9 ± 7.4	9.6 ± 7.8	12.3 ± 7.9	8.7 ± 6.6	**0.03**
ESS	7.7 ± 5.6	7.4 ± 5.8	7 ± 6.3	8.3 ± 5.1	0.197
MoCA	23.6 ± 4.9	23.1 ± 4.2	22.8 ± 5.7	24.3 ± 4.6	0.061
HAMA	9.1 ± 7.1	9.6 ± 8.4	10.9 ± 8.1	7.9 ± 5.6	0.15
HAMD-17	5.8 ± 5.2	6.4 ± 5.7	7 ± 6.1	4.9 ± 4.3	0.171

*Data are presented as number (%) or as mean ± standard deviation, as appropriate. The number in bold indicates significance (p < 0.05)*.

*BMI, body mass index; TST, total sleep time; REM, rapid eye movement; N1, non-REM (NREM) stage 1; N2, non-REM (NREM) stage 2; N3, non-REM (NREM) stage 3; Mean SpO_2_, Mean oxygen saturation; Lowest SpO_2_, Lowest oxygen saturation; ISS, Insomnia Severity Index; ESS, Epworth Sleepiness Scale; MoCA, Montreal Cognitive Assessment; HAMA, Hamilton Anxiety Scale; HAMD-17, Hamilton Depression Scale-17*.

### Characteristics of OSA Patients With Different Severity

306 patients received PSG in the laboratory, and all patients had sleep apnea. Divide the patients into three groups according to AHI: 69 cases were mild (5 ≤ AHI < 15), 86 cases were moderate (15 ≤ AHI < 30), and 151 cases were severe (AHI ≥ 30). Age, smoking history, sleep efficiency, rapid eye movement accounted for total sleep time (REM%) and most scale scores were not statistically different among OSA patients of different severity (all *P* > 0.05). The post-examination found that compared with patients in the mild and moderate groups, the severe group had more male patients, higher body mass index (BMI) and non-REM stage 1 accounted for total sleep time (N1%), and lower non-REM stage 2 accounted for total sleep time (N2%), average and minimum blood oxygen.

### The Status of Receiving Titration Test

63 patients accepted titration test, and 243 patients were not treated (Table 2). Although gender and minimum blood oxygen saturation were found to be significantly related to AHI, multicollinearity was not serious. Therefore, age, gender, educational background, sleep efficiency, AHI, and minimum blood oxygen saturation were included in the logistic regression model. The dependent variable is divided into accepting titration test and not accepting titration test. Multivariate analysis showed that sleep efficiency (OR = 1.043, 95%CI: 1.02–1.067, *P* < 0.001) and AHI (OR = 1.022, 95%CI: 1.01–1.034, *P* < 0.001) were independent factors related to titration test in patients with OSA and hypertension (Table 3).

**Table 2:** Characteristics of the patients according to the treatments for OSA.

**Characteristics**	**Total cohort**	**Titration test**	**No treatment**	***p*-value**
	**(*n =* 306)**	**(*n =* 63)**	**(*n =* 243)**	
**Demographic and clinical characteristics**				
% of total	100	20.6	79.4	
Age, *y*	55.8 ± l2.9	51.5 ± 12.1	56.9 ± 12.9	**0.005**
Male, *n* (%)	213 (69.6)	51 (81)	162 (66.7)	**0.028**
BMI, kg/m^2^	27.1 ± 4.3	27.8 ± 4.7	26.9 ± 4.2	0.221
Tobacco use, *n* (%)	139 (45.4)	30 (47.6)	109 (44.9)	0.695
Alcohol drinking, *n* (%)	170 (55.6)	38 (60.3)	132 (54.3)	0.393
Education background, *n* (%)				**0.045**
≤9 years	116 (37.9)	17 (27)	99(40.7)	
>9 years	190 (62.1)	46 (73)	144 (59.3)	
**Polysomnographic data**				
Sleep efficiency, %	70.3 ± 16.5	77.2 ± 13.6	68.6 ± 16.8	**<0.001**
**Sleep stage, %TST**				
REM	15.7 ± 6.8	15.5 ± 5.3	15.8 ± 7.1	0.877
N1	25 ± 17.1	24.2 ± 15.5	25.2 ± 17.6	0.93
N2	54.2 ± 15.3	55.4 ± 14.1	53.9 ± 15.6	0.615
N3	5 ± 6.2	4.9 ± 5.3	5.1 ± 6.4	0.881
AHI, events/h	34.9 ± 23.5	44.7 ± 25.6	32.3 ± 22.2	**<0.001**
Mean SpO_2_, %	94.6 ± 2.9	94.4 ± 3.3	94.7 ± 2.8	0.466
Lowest SpO_2_, %	81.4 ± 9.6	78.8 ± 11.7	82.1 ± 8.8	**0.038**
**Scale**				
ISI	9.9 ± 7.4	10 ± 7.3	9.9 ± 7.4	0.95
ESS	7.7 ± 5.6	7.63 ± 4.7	7.8 ± 5.9	0.854
MoCA	23.6 ± 4.9	24.6 ± 3.6	23.4 ± 5.1	0.265
HAMA	9.1 MA5.1	8.9 MA5.4	9.2 MA5.3	0.82
HAMD-17	5.8 ± 5.2	6 ± 5.6	5.7 ± 5.1	0.924

*Data are presented as %, unless otherwise stated. Bold font indicates variables with statistically significant differences between clusters (p < 0.05)*.

*BMI, body mass index; TST, total sleep time; REM, rapid eye movement; N1, non-REM (NREM) stage 1; N2, non-REM (NREM) stage 2; N3, non-REM (NREM) stage 3; AHI, apnoea-hypopnoea index; Mean SpO_2_, Mean oxygen saturation; Lowest SpO_2_, Lowest oxygen saturation; ISS, Insomnia Severity Index; ESS, Epworth Sleepiness Scale; MoCA, Montreal Cognitive Assessment; HAMA, Hamilton Anxiety Scale; HAMD-17, Hamilton Depression Scale-17*.

**Table 3:** Multivariate analyse of independent factors associated with the decisions for titration treatment-test.

**Variable**	**Multivariate analysis**		
	**OR**	**95%CI**	***p*-value**
Age			0.595
Sex			0.213
Education background			0.123
Sleep efficiency	1.043	1.02–1.067	**<0.001**
AHI	1.022	1.01–1.034	**<0.001**
Lowest SpO_2_			0.629

*The number in bold indicates significance (p < 0.05)*.

*AHI, apnoea-hypopnoea index; Lowest SpO_2_, Lowest oxygen saturation*.

## Discussion

This study is the first to investigate the sleep structure and treatment status of OSA patients with hypertension. It was found that severe group had a higher BMI and N1%, and a lower N2%. In addition, patients with OSA and hypertension have low acceptance of titration tests. The results showed that only 20.6% of the 306 patients underwent the titration test. Multivariate analysis showed that AHI and sleep efficiency were independent factors for OSA patients to undergo titration tests.

In a longitudinal study of weight change and sleep-disordered breathing, baseline BMI was a significant predictor of AHI changes. For every 1 kg/m^2^ added in baseline BMI, AHI was expected to increase by about 1% (21). During sleep in OSA patients, complete or partial obstruction of the upper airway occurs repeatedly, often accompanied by decreased blood oxygen saturation and arousal. The continuous process of sleep is interrupted to form fragmented sleep. Issa and Sullivan found that OSA patients were more likely to collapse in the upper airway during light sleep stage when they evaluated the upper airway obstruction technique (22). Therefore, compared with mild and moderate patients, severe patients have more apneas, and then N1% will be higher.

The apnea that occurs during sleep in OSA patients can cause hypoxia and high carbon dioxide partial pressure, stimulate peripheral and central chemoreceptors, re-regulate the blood pressure threshold, and develop essential hypertension. In addition, oxidative stress and inflammation are also the mechanisms that can cause hypertension in patients with OSA (23, 24).

A team has found that OSA and hypertension have an interactive effect on the progression of carotid atherosclerosis and the level of inflammatory markers of atherosclerosis in the blood (such as interleukin-6 and pentapeptidase-3). Compared with OSA patients with normal blood pressure, hypertension patients without OSA or the control group, the carotid artery intima-media thickness and early markers of atherosclerosis were significantly increased (25, 26). Therefore, OSA patients with hypertension often have more serious target organ damage. This kind of crowd should be highly valued by doctors and be given early treatment intervention.

The American Academy of Sleep Medicine recommends that clinicians use CPAP to treat adult OSA patients with hypertension (12). Studies have confirmed that in patients with hypertension and OSA, after long-term use of CPAP standardized treatment, daytime systolic blood pressure is significantly reduced, and daytime sleepiness has also been improved (27).

However, the treatment condition is not ideal. In the past 20 years, the overall non-compliance rate of CPAP was 34.1% (28). Many previous studies have focused on compliance with CPAP treatment, but about 5–50% of patients who were recommended to use CPAP refused this treatment or stopped treatment within the 1st week (29). Therefore, the acceptance of CPAP treatment should also be considered. CPAP pressure titration test is an important link that would affect patients' subsequent treatment. Previous studies investigated the reasons why patients did not receive CPAP treatment, including patients thought they did not need treatment, did not know how to treat them, lacked knowledge of the disease, and believed that the machine was inconvenient to use. But even if patients are given standardized consultation before APAP titration, the acceptance of CPAP by OSA patients is still very low (19).

A previous study on elderly OSA patients showed that compared with mild patients, moderate AHI patients and severe patients are more likely to receive CPAP (18). Our study also reached a similar conclusion, and found that AHI is an independent factor in the titration test for patients with OSA and hypertension. Although previous studies have pointed out that the ESS score is an important predictor of CPAP acceptance, our study did not find an association between ESS score and acceptance. Some studies have reported that the accuracy of this scale was dependent on the participants' gender, psychological variables and subjective perception (30–32). Therefore, it is controversial to take ESS score as a predictor of acceptance. Previous studies have also found that lower CPAP acceptance is related to living alone, mild symptoms (especially drowsiness), less skills, impaired cognitive ability, comorbidities, and neurological deficits (33). Economic incentives can increase the acceptance of CPAP by low-income patients (34). However, these conclusions have not been confirmed in this study, so multi-center, large sample size and more comprehensive studies are needed to verify in the future.

For the first time our study found that sleep efficiency is an independent factor in the titration test for patients with OSA and hypertension. Sleep efficiency itself is affected by many factors. A laboratory PSG-based study assessed patients' sleep quality and explored factors related to poor sleep. In a multivariate analysis, it was found that older, male, and severe OSA patients are more likely to suffer from sleep deprivation (35). However, this study found that the multicollinearity between sleep efficiency and the variables mentioned above is not serious, so all of them can be included in the regression model together. In addition, because the PSG in the laboratory has a “first night effect”, it will greatly reduce the patient's sleep efficiency (36). However, there was no significant difference in sleep efficiency between the PSG laboratory group and the home monitoring group (37). If the “first night effect” of patients with OSA and hypertension can be reduced, and their sleep efficiency can be improved, then their acceptance of the CPAP titration test can be boosted.

This study still has certain limitations. First, the sample size was relatively small and came from a single center. Furthermore, the retrospective design prevented the investigation of the patients' specific blood pressure at that time. For OSA patients, especially those with hypertension, CPAP treatment is the key to improving the quality of life, reducing accidents and improving cognitive outcomes (38). Successfully promoting the use of CPAP for patients depends on the medical team's connection and problem solving in each step of the therapeutic process. More research is necessary to determine ways to improve patients' acceptance of CPAP.

## Conclusion

In summary, it can be found that a large proportion of OSA patients with hypertension do not receive correct treatment guidance, and the rate of receiving a titration test is at a low level. Patients with higher AHI and higher sleep efficiency are more likely to undergo a titration test. In the future, more efforts are needed to solve these patients' understanding to the disease, and more research is needed to comprehensively explore the factors that influence patients' treatment decision. In this way, we could draw up personalized and optimal treatment protocols for these people who are in urgent need of treatment.

## Data Availability Statement

The raw data supporting the conclusions of this article will be made available by the authors, without undue reservation.

## Ethics Statement

The studies involving human participants were reviewed and approved by the Medical Ethics Committee of Beijing Tiantan Hospital and the Medical Ethics Committee of Capital Medical University. The patients/participants provided their written informed consent to participate in this study.

## Author Contributions

XZ and C-XW designed the study. XZ, C-XW, NZ, YY, SW, PY, and B-YG collected and analyzed the data. PY and B-YG interpreted the results. XZ drafted the manuscript. XZ, YY, and SW were major contributors in writing and editing the manuscript. All authors read, revised, and approved the final manuscript.

## Funding

This study was funded by National Key Research & Development Program of China (No. 2020YFC2005304) and Capital's Funds for Health Improvement and Research (2020-2-2044).

## Conflict of Interest

The authors declare that the research was conducted in the absence of any commercial or financial relationships that could be construed as a potential conflict of interest.

## Publisher's Note

All claims expressed in this article are solely those of the authors and do not necessarily represent those of their affiliated organizations, or those of the publisher, the editors and the reviewers. Any product that may be evaluated in this article, or claim that may be made by its manufacturer, is not guaranteed or endorsed by the publisher.
